# Reviewed article: Curado DSP, Silva EV. Drugs incorporated into the Brazilian Unified Health System: documentary analysis of methodological decisions in budget impact analyses between 2012 and 2024. Epidemiol Serv Saude. 2025:34;e20250122.

**DOI:** 10.1590/S2237-96222025v34e20250122.a

**Published:** 2025-09-08

**Authors:** Marcos Lima Almeida

**Affiliations:** Universidade Federal do Piaui

**Table:** 

Rating	Excellent	Good	Average	Below average	Poor
Interest		✓			
Quality	✓				
Originality	✓				
Overall	✓				

**Table:** 

Questionnaire	Yes	No	Not applicable
Does the manuscript contain new and significant information to justify publication?	✓		
Does the Abstract (Summary) clearly and accurately describe the content of the article?	✓		
Is the problem significant and concisely stated?	✓		
Are the methods described comprehensively?	✓		
Are the interpretations and conclusions justified by the results?	✓		
Is adequate reference made to other work in the field?	✓		

## Manuscript Structure

Length of article is: Adequate

Number of tables is: Adequate

Number of figures is: Too little

Please state any conflict(s) of interest that you have in relation to the review of this paper (state “none” if this is not applicable): None

## Comments

O estudo é uma contribuição fundamental para a compreensão de como o SUS lida com a incorporação de novos medicamentos e como as análises de impacto orçamentário podem ser aprimoradas. Ele revela que, apesar do avanço nas metodologias, ainda existem pontos críticos a serem resolvidos, como a consistência na coleta de dados e a abordagem mais abrangente dos custos. A incorporação de medicamentos é uma questão importante para garantir o acesso à saúde de qualidade, mas também precisa ser financeiramente viável para garantir a sustentabilidade do sistema de saúde pública no Brasil. Parabéns pelo estudo, ficou muito bom. 

